# Prematurity and Low Birth Weight did not Correlate with Anti-*Toxoplasma gondii* Maternal Serum Profiles – a Brazilian Report

**DOI:** 10.1371/journal.pone.0132719

**Published:** 2015-07-20

**Authors:** Mariana Machado Lemos Fochi, Sabrina Baring, Lígia Cosentino Junqueira Franco Spegiorin, Denise Cristina Mós Vaz-Oliani, Eloisa Aparecida Galão, Antonio Hélio Oliani, Luiz Carlos de Mattos, Cinara Cássia Brandão de Mattos

**Affiliations:** 1 Immunogenetics Laboratory, Department of Molecular Biology, Faculdade de Medicina de São José do Rio Preto–FAMERP, São José do Rio Preto, São Paulo, Brazil; 2 Obstetrics and Gynecology Service, Hospital de Base, Fundação Faculdade Regional de Medicina de São José do Rio Preto–HB-FUNFARME, São José do Rio Preto, São Paulo, Brazil; 3 Department of Gynecology and Obstetrics, Faculdade de Medicina de São José do Rio Preto–FAMERP, São José do Rio Preto, São Paulo, Brazil; 4 Hospital da Criança e Maternidade de São José do Rio Preto–HCM, São José do Rio Preto, São Paulo, Brazil; 5 FAMERP Toxoplasma Research Group, Faculdade de Medicina de São José do Rio Preto–FAMERP, São José do Rio Preto, São Paulo, Brazil; Institut national de la santé et de la recherche médicale - Institut Cochin, FRANCE

## Abstract

Gestational *Toxoplasma gondii* infection is considered a major risk factor for miscarriage, prematurity and low birth weight in animals. However, studies focusing on this topic in humans are scarce. The objective of this study is to determine whether anti-*Toxoplasma gondii* maternal serum profiles correlate prematurity and low birth weight in humans. The study examined 213 pregnant women seen at the High-Risk Pregnancy Hospital de Base, São José do Rio Preto, São Paulo, Brazil. All serological profiles (IgM-/IgG+; IgM-/IgG-; IgM+/IgG+) were determined by ELISA commercial kits. Maternal age, gestational age and weight of the newborn at birth were collected and recorded in the Statement of Live Birth. Prematurity was defined as gestational age <37 weeks and low birth weight ≤ 2499 grams. The *t*-test was used to compare values (p < 0.05). The mean maternal age was 27.6±6.6 years. Overall, 56.3% (120/213) of the women studied were IgM-/IgG+, 36.2% (77/213) were IgM-/IgG- and 7.5% (16/213) were IgM+/IgG+. The average age of the women with serological profile IgM+/IgG+ (22.3±3.9 years) was different from women with the profile IgM-/IgG+ (27.9±6.7 years, p = 0.0011) and IgM-/IgG- (27.9±6.4 years, p = 0.0012). There was no statistically significant difference between the different serological profiles in relation to prematurity (p = 0.6742) and low birth weight (p = 0.7186). The results showed that prematurity and low birth weight did not correlate with anti-*Toxoplasma gondii* maternal serum profiles.

## Introduction

*Toxoplasma gondii* is an obligate intracellular parasite that causes toxoplasmosis, one of the most widespread zoonotic diseases in the world. In all countries there is a high number of humans and animals infected with *T*. *gondii*. In some regions, about 30–60% of the population is positive for toxoplasmosis in serological tests [1–4].

The *T*. *gondii* life cycle alternates between intermediate hosts (mammals and birds), where the asexual stage occurs, and definitive hosts (felines), harboring the sexual stage. The infection in the intermediate host occurs by eating raw or undercooked meat containing cysts, and water or food contaminated with oocysts secreted in the feces of infected cats [2].

In the acute phase, rapid replication of tachyzoites predominates and in approximately 60–90 days the infection becomes chronic. As the immune response is effective in controlling the infection, tachyzoites differentiate into bradyzoites, which divide more slowly and form cysts in various cells, particularly in the brain, heart and muscles. About 80% of chronically infected individuals are asymptomatic although in some cases eye injuries occur [2,5–11].

When the primary infection occurs during pregnancy, the parasites can infect the fetus through the placenta, causing birth defects and ocular complications. The consequences of maternal and fetal infection depend on the degree of exposure of the fetus to parasites, the virulence of the strain and the gestational period in which there was infection, and the classic signs of congenital toxoplasmosis are: hydrocephalus; chorioretinitis; cranial calcifications and mental retardation [1,12–23].

Contemporary research on *T*. *gondii* infection in humans has been directed to risk groups such as patients with immunodeficiency, transplant patients, patients with eye injuries and even normal individuals. In addition to these, pregnant women and newborns are targeted for medical attention given the risks of congenital transmission and the sequelae [14,24–30].

The prevalence of *T*. *gondii* infection was investigated in different Brazilian states in recent decades and the results revealed great variability in its contents, including previous studies by our group [2,31–34]. In addition to these studies, we were able to establish that the congenital transmission rate in the region reaches 2.3% [32]. The interest in conducting proper screening for *T*. *gondii* infection in risk groups has grown considerably in recent years, as well as the interest in studies to establish some relation to the conditions presented by newborns, such as prematurity and disease severity [5,18,35–42].

Toxoplasmosis thus constitutes a serious public health problem, especially in pregnant women who were not infected by *T*. *gondii*, and may cause neurologic damage the baby, being a disease of epidemiological significance to pregnant women and women of childbearing age and recently included in the list of by the CDC of neglected diseases [43,44].

The aim of this study was to correlate prematurity and low birth weight with the serological profile of pregnant women for toxoplasmosis.

## Materials and Methods

### Ethical aspects of the study

This study was approved by the Ethics Committee in Research of the Faculty of Medicine of São José do Rio Preto–FAMERP (protocol 168/2007). The need for a written consent of patients was waivered as all the data were retrospectively collected from the patients' hospital records and was anonymized.

### Data analysis

Data records were consulted for 310 pregnant women who met at the Clinic of High Risk Pregnancy and Fetal Medicine from the Hospital de Base de São José do Rio Preto, Brazil from 2005 to 2007. Complete data were found for 213 women regarding serological screening for toxoplasmosis during the prenatal period; weight data, gestational age and maternal age were collected from their Statement of Live Birth.

### Definition of prematurity and birth weight

Preterm birth was defined as gestational age less than 37 weeks and low birth weight as less than or equal to 2499 grams according to the criteria of the World Health Organization [45].

### Definition of serological profiles for toxoplasmosis

All serological profiles were determined by ELISA commercial kits (Diasorin, Italy) and all manufacturer’s instructions were strictly followed. Three serological profiles were constructed: **I** = IgM positive/ IgG positive; **II** = IgM negative/IgG positive; **III** = IgM negative/IgG negative. The profile IgM positive/IgG negative was not found in the analyzed cases. All maternal serum profiles were identified at the first medical consultation.

### Statistical analysis

The *t*-test was used to compare the data according to the serological profiles. The alpha value less than or equal to 5% was considered significant.

## Results

The average age of the selected pregnant women was 27.6±6.6 years. The results are shown in Tables 1, 2 and 3. In Table 1, it can be seen that the profile IgM negative/IgG positive (Profile II) prevails over the other.

**Table 1:** Serological profile of pregnant women seen at the Clinic of High Risk Pregnancy and Fetal Medicine of Hospital de Base de São José do Rio Preto, São Paulo, Brazil from 2005 to 2007.

Profile	Serology	N	%
**I**	IgM+/IgG+	16	7.5
**II**	IgM-/IgG+	120	56.3
**III**	IgM-/IgG-	77	36.2
	**Total**	**213**	

(+) = positive; (-) = negative.

**Table 2:** Comparison of serological profile for anti-*T*. *gondii* antibodies and maternal age.

Profile	Serology	N	Average Maternal Age
**I**	IgM+/IgG+	16	22.3 ± 3.9
**II**	IgM-/IgG+	120	27.9 ± 6.7
**III**	IgM-/IgG-	77	27.9 ± 6.4
	**Total**	**213**	

I x II: p = 0.0011; I x III: p = 0.0012; II x III: p = 0.9999

**Table 3:** Relationship between low birth weight and maternal serological profile serological profile for anti-*T*. *gondii* immunoglobulins.

Profile	Serology	Low Birth Weight		Non Low Birth Weight	
		N	%	N	%
**I**	IgM+/IgG+	02	12.5	14	87.5
**II**	IgM-/IgG+	25	20.8	95	79.2
**III**	IgM-/IgG-	18	23.4	59	76.6
	**Total**	**45**		**168**	

I x II: p = 0.7384; I x III: p = 0.5078; II x III: p = 0.7250

Table 2 shows the average age of pregnant women according to the identified serologic profiles. No statistically significant differences were observed between the mean age of the women with the profiles II and III (p = 0.9999). However, the average age of the women in profile I was lower than those observed for the women in profile II (p = 0.0011) and III (p = 0.0012).

The average birth weight of newborns analyzed was 1,916 ± 617.1 grams. Table 3 lists the maternal serological profile and weight of the newborn. No statistically significant differences were observed between the different serological profiles (I, II and III) and the low weight of the newborn (I x II: p = 0.7384; I x III: p = 0.5078; II x III: p = 0.7250).

The mean gestational age was 33.7 ± 3.7 weeks. Table 4 shows the relationship between the serological profile and prematurity. Profiles I, II and III when compared showed no statistically significant difference (I x II: p = 0.7780; I x III: p = 0.7681; II x III: p = 0.7534). Table 5 shows the frequency of birth weight classifications.

**Table 4:** Relationship between prematurity and maternal serological profile for anti-*T*. *gondii* immunoglobulins.

Profile	Serology	Prematurity		Non-Prematurity	
		N	%	N	%
**I**	IgM+/IgG+	04	25	12	75
**II**	IgM-/IgG+	36	30	84	70
**III**	IgM-/IgG-	25	32.4	52	67.6
	**Total**	**65**		**148**	

I x II: p = 0.7780; I x III: p = 0.7681; II x III: p = 0.753

**Table 5:** Frequency of weight at birth and serological profiles for *T*. *gondii*, São José do Rio Preto, São Paulo.

Serology	Normal Weight >2.500g	Low Birth Weight <2.500g	Very Low Birth Weight <1.500g	Extremely Low Birth Weight <1.000g	Total
**IgM-/IgG-**	55	19	1	2	77
**IgM-/IgG+**	99	16	2	3	120
**IgG+/IgM+**	14	1	0	1	16
**Total**	**168**	**36**	**3**	**6**	**213**

## Discussion

The objective of this study was to correlate prematurity and low birth weight with the maternal serum profiles in 213 pregnant women for toxoplasmosis the northwestern region of São Paulo State who gave birth a single baby for both sexes, whose data prematurity and low birth were obtained from the official Statement of Live Birth. The decision to use this document as a data source is due to the fact that they constitute an official record established by Brazilian legislation. [41,42,46].

The frequencies of the identified serological profiles in patients in our study are consistent with findings in the literature [47,48]. The prevalence of *T*. *gondii* infection is high in the northwest of São Paulo State and it has been shown that most pregnant women have the anti-*T*. *gondii* IgG class. Even so, a large percentage of individuals do not present specific antibodies for this parasite and this may result from having contact with the infective form. The simultaneous presence of anti-*T*. *gondii* IgM and IgG classes is transient in most cases and therefore the percentage of pregnant women with this profile is smaller than the other mentioned above [14,18,31,47,49,50].

The results showed that the pregnant women’s positive containing only anti-*Toxoplasma* IgG antibodies (profile II) have an average age of pregnant women as the negative for both classes of antibodies (profile III). However, the mean age of the women with these serological profiles was higher than that of positive pregnant women for both antibody classes (profile I).

There are reports that the average age of pregnant women at increased risk of congenital infection (IgM+/IgG- and IgM+/IgG+) is lower than those with other serological profiles (IgM-/IgG- and IgM-/IgG+). Pessanha et al. [51] reported that the average age of women who gave birth to children with laboratory indications of congenital infection was lower than those whose newborns did not present signs of infection. Although the mean age of the pregnant women with serological profile I was lower in comparison with those carrying serological profile II and III; we must take in account that IgM+/IgG+ serum profile does not confer risk of congenital toxoplasmosis transmission.

Dias et al. [52], Rodrigues et al. [47] and Ferenzi and et al. [53] reported a higher mean age for positive pregnant women with IgG anti-*T*. *gondii* compared to those not exposed to the parasite. These observations are supported by the data observed in this study. In fact, chronic infection maintenance and the risk of reinfection contribute to the production of IgG class antibodies and with a higher avidity index [1,4,14,54–56]. The high rates of infection in the region of origin of pregnant women evaluated in this study can contribute to re-exposure to the parasite favoring the prevalence of anti-*T*. *gondii* IgG class antibodies in the population [2,31,47,57–60].

The average age of pregnant women with serological profile III did not differ from that observed in women with pattern II and this can be a result that the series were selected in a reference center caring for high-risk pregnant women.

This study also explored the relationships between serum maternal profile with low-birth and prematurity since these variables were previously reported [5,30,40–42,59–64]. A report from Moraes et al. [65] showed that the prevalence of low-birth and prematurity for the region in which this study was carried out is equal to 6.5% and 9.0%, respectively. These values are lower in comparison with our data since the medical records were collected from a reference center for high risk pregnancy.

Comparison of birth weight with different serological profiles did not show statistically significant differences. Pessanha et al. [51] observed that birth weight is not correlated with the presence or absence of congenital infection by *T*. *gondii*. Although Pessanha did not correlate serological profiles of pregnant women with birth weight, their data underlie, at least in part, the results reported in this study.

Prematurity observed in the analyzed sample showed no correlations with the maternal serum profiles found. McLeod et al. found that serotypes of *T*. *gondii* NE II are associated with prematurity and/or severity of congenital toxoplasmosis but did not establish correlations of these conditions to the serological profiles of the analyzed pregnant women. [36]

This study present some limitations and therefore their data must be considered as preliminary. First, only ELISA test was performed to detect IgM and IgG anti-*T*. *gondii* antibodies and it present some limitations. Persistent IgM antibodies can be detected for several months after the primary infection and therefore IgM+/IgG+ serum profile does not correlate acute infection necessarily [1,50,56,66,67]. Secondly, IgG avidity test and Western blot test were not carried out in the pregnant women and their neonates since they are not offered by Brazilian public health system [14,39,68–70]. Studies with large numbers of pregnant women in different Brazilian regions may contribute to the elucidation of the importance of analysis of maternal serum profiles and their potential correlations with low birth weight and prematurity and infection by *T*. *gondii*.n conclusion, our data show that maternal age is related to the serological profile, but prematurity and low birth weight did not correlate with anti-*T*. *gondii* maternal serum profiles.

## References

[1] Robert-Gangneux F, Dardé MM-L. Epidemiology of and diagnostic strategies for toxoplasmosis. Clinical microbiology reviews [Internet]. 2012 4 [cited 2014 Oct 10];25(2):264–96. Availablew: http://www.scopus.com/inward/record.url?eid=2-s2.0-84859523382&partnerID=tZOtx3y1. 10.1128/CMR.05013-11 22491772 PMC3346298

[2] Dubey JP, Lago EG, Gennari SM, Su C, Jones JL. Toxoplasmosis in humans and animals in Brazil: high prevalence, high burden of disease, and epidemiology. Parasitology [Internet]. 2012 9 [cited 2014 Oct 28];139(11):1375–424. Available: http://www.scopus.com/inward/record.url?eid=2-s2.0-84870152160&partnerID=tZOtx3y1. 10.1017/S0031182012000765 22776427

[3] Pappas G, Roussos N, Falagas ME. Toxoplasmosis snapshots: global status of Toxoplasma gondii seroprevalence and implications for pregnancy and congenital toxoplasmosis. International journal for parasitology [Internet]. 2009 10 [cited 2014 Sep 30];39(12):1385–94. Available: http://www.ncbi.nlm.nih.gov/pubmed/19433092. 10.1016/j.ijpara.2009.04.003 19433092

[4] Lopes AP, Dubey JP, Dardé M-L, Cardoso L. Epidemiological review of Toxoplasma gondii infection in humans and animals in Portugal. Parasitology [Internet]. Cambridge University Press; 2014 11 1 [cited 2014 Dec 21];141(13):1699–708. Available: http://journals.cambridge.org/abstract_S0031182014001413. 10.1017/S0031182014001413 25215422

[5] Wallon M, Garweg JG, Abrahamowicz M, Cornu C, Vinault S, Quantin C, et al Ophthalmic outcomes of congenital toxoplasmosis followed until adolescence. Pediatrics [Internet]. 2014 3 1 [cited 2014 Nov 30];133(3):e601–8. Available: http://www.ncbi.nlm.nih.gov/pubmed/24534412. 10.1542/peds.2013-2153 24534412

[6] Ferreira AIC, De Mattos CCB, Frederico FB, Meira CS, Almeida GC, Nakashima F, et al Risk factors for ocular toxoplasmosis in Brazil. Epidemiology and infection [Internet]. Cambridge University Press; 2014 1 1 [cited 2014 Oct 28];142(1):142–8. Available: http://journals.cambridge.org/abstract_S0950268813000526. 10.1017/S0950268813000526 23507508 PMC3857107

[7] Furtado JM, Mph KLW, Butler NJ, Mbbs JRS, Gondii T. Review Ocular toxoplasmosis I: parasitology, epidemiology and public health OF. 2012;(February). 10.1111/j.1442-9071.2012.02821.x 22594908

[8] Pleyer U, Schlüter D, Mänz M. Ocular Toxoplasmosis: Recent Aspects of Pathophysiology and Clinical Implications. Ophthalmic research [Internet]. Karger Publishers; 2014 1 [cited 2014 Dec 21];52(3):116–23. Available: http://www.karger.com/Article/FullText/363141. 10.1159/000363141 25248050

[9] Munoz-Zanzi CA, Fry P, Lesina B, Hill D. Toxoplasma gondii oocyst-specific antibodies and source of infection. Emerging infectious diseases [Internet]. 2010 10 [cited 2014 Dec 21];16(10):1591–3. Available: http://www.pubmedcentral.nih.gov/articlerender.fcgi?artid=3294384&tool=pmcentrez&rendertype=abstract. 10.3201/eid1610.091674 20875286 PMC3294384

[10] Asilsoy S, Babayigit A, Olmez D, Uzuner N, Karaman O, Oren O, et al Helicobacter pylori infection and gastroesophageal reflux in asthmatic children. Journal of tropical pediatrics [Internet]. 2008 4 1 [cited 2014 Nov 30];54(2):129–32. Available: http://www.ncbi.nlm.nih.gov/pubmed/18039679. 18039679 10.1093/tropej/fmm069

[11] Sullivan WJ, Jeffers V WS Jr, Jeffers V, Sullivan WJ, Jeffers V. Mechanisms of Toxoplasma gondii persistence and latency. FEMS microbiology reviews [Internet]. 2012 5 [cited 2014 Dec 21];36(3):717–33. Available: http://www.pubmedcentral.nih.gov/articlerender.fcgi?artid=3319474&tool=pmcentrez&rendertype=abstract. 10.1111/j.1574-6976.2011.00305.x 22091606 PMC3319474

[12] Garcia AGP, Coutinho SG, Amenodeira MRR, Assumpção MR, Albano N. Placental Morphology of Newborns at Risk for Congenital Toxoplasmosis. Journal of Tropical Pediatrics [Internet]. 1983 4 1 [cited 2014 Nov 30];29(2):95–103. Available: http://tropej.oxfordjournals.org/cgi/doi/10.1093/tropej/29.2.95. 6854713 10.1093/tropej/29.2.95

[13] Segundo GRS. Congenital Toxoplasmosis in Uberlandia, MG, Brazil. Journal of Tropical Pediatrics [Internet]. 2004 2 1 [cited 2014 Nov 30];50(1):50–3. Available: http://tropej.oupjournals.org/cgi/doi/10.1093/tropej/50.1.50. 14984171 10.1093/tropej/50.1.50

[14] Rodrigues IIM, Costa TLT, Avelar JB, Amaral WN, Castro AM, Avelino MM. Assessment of laboratory methods used in the diagnosis of congenital toxoplasmosis after maternal treatment with spiramycin in pregnancy. BMC infectious diseases [Internet]. BioMed Central Ltd.; 2014 1 [cited 2014 Nov 3];14(1):349 Available: http://www.biomedcentral.com/1471-2334/14/349/. 24961630 10.1186/1471-2334-14-349 PMC4230641

[15] Mario S Di, Basevi V. Prenatal education for congenital toxoplasmosis. … Database Syst Rev [Internet]. 2009 [cited 2014 Dec 31];(2). Available: http://onlinelibrary.wiley.com/doi/10.1002/14651858.CD006171.pub2/pdf/standard.

[16] De Jong EP, Vossen ACTM, Walther FJ, Lopriore E. How to use… neonatal TORCH testing. Archives of disease in childhood Education and practice edition [Internet]. 2013 6 [cited 2014 Oct 24];98(3):93–8. Available: http://www.ncbi.nlm.nih.gov/pubmed/23470252. 10.1136/archdischild-2012-303327 23470252

[17] Mandelbrot L. Toxoplasmosis y embarazo. EMC—Ginecología-Obstetricia [Internet]. 2014 12 [cited 2014 Dec 21];50(4):1–12. Available: http://www.sciencedirect.com/science/article/pii/S1283081X14692870.

[18] Stillwaggon E, Carrier CS, Sautter M, McLeod R. Maternal serologic screening to prevent congenital toxoplasmosis: a decision-analytic economic model. PLoS neglected tropical diseases [Internet]. 2011 9 [cited 2014 Dec 21];5(9):e1333 Available: http://www.pubmedcentral.nih.gov/articlerender.fcgi?artid=3181241&tool=pmcentrez&rendertype=abstract. 10.1371/journal.pntd.0001333 21980546 PMC3181241

[19] Collantes-Fernandez E, Arrighi RBG, Alvarez-García G, Weidner JM, Regidor-Cerrillo J, Boothroyd JC, et al Infected dendritic cells facilitate systemic dissemination and transplacental passage of the obligate intracellular parasite Neospora caninum in mice. PloS one [Internet]. 2012 1 [cited 2014 Dec 17];7(3):e32123 Available: http://www.pubmedcentral.nih.gov/articlerender.fcgi?artid=3293873&tool=pmcentrez&rendertype=abstract. 10.1371/journal.pone.0032123 22403627 PMC3293873

[20] Herrmann DC, Maksimov P, Hotop A, Groß U, Däubener W, Liesenfeld O, et al Genotyping of samples from German patients with ocular, cerebral and systemic toxoplasmosis reveals a predominance of Toxoplasma gondii type II. International journal of medical microbiology: IJMM [Internet]. 2014 10 [cited 2014 Dec 21];304(7):911–6. Available: http://www.sciencedirect.com/science/article/pii/S1438422114000770. 10.1016/j.ijmm.2014.06.008 25037927

[21] Pardini L, Carral LA, Bernstein M, Gos ML, Olejnik P, Unzaga JM, et al First isolation and molecular characterization of Toxoplasma gondii from a human placenta in Argentina. Parasitology international [Internet]. 2014 4 [cited 2014 Dec 21];63(2):470–2. Available: http://www.sciencedirect.com/science/article/pii/S1383576913001712. 10.1016/j.parint.2013.10.011 24513795

[22] Carneiro ACAV, Andrade GM, Costa JGL, Pinheiro BV, Vasconcelos-Santos DV, Ferreira AM, et al Genetic characterization of Toxoplasma gondii revealed highly diverse genotypes for isolates from newborns with congenital toxoplasmosis in southeastern Brazil. Journal of clinical microbiology [Internet]. 2013 3 1 [cited 2014 Dec 21];51(3):901–7. Available: http://www.pubmedcentral.nih.gov/articlerender.fcgi?artid=3592078&tool=pmcentrez&rendertype=abstract. 10.1128/JCM.02502-12 23284022 PMC3592078

[23] Silva LLA, Andrade RRO, Carneiro ACA V, Vitor RRWA. Overlapping Toxoplasma gondii Genotypes Circulating in Domestic Animals and Humans in Southeastern Brazil. PloS one [Internet]. Public Library of Science; 2014 1 27 [cited 2014 Dec 21];9(2):e90237 Available: http://dx.plos.org/10.1371/journal.pone.0090237.g001. 10.1371/journal.pone.0090237 PMC3937362 24587295

[24] Fernàndez-Sabé N, Cervera C, Fariñas MC, Bodro M, Muñoz P, Gurguí M, et al Risk factors, clinical features, and outcomes of toxoplasmosis in solid-organ transplant recipients: a matched case-control study. Clinical infectious diseases: an official publication of the Infectious Diseases Society of America [Internet]. 2012 2 1 [cited 2014 Dec 21];54(3):355–61. Available: http://cid.oxfordjournals.org/content/54/3/355.short. 22075795 10.1093/cid/cir806

[25] González-Padilla M, Castón JJ, Vidal E, Arizón JM, Segura C, Montejo M, et al Epidemiology and clinical impact of infection in patients awaiting heart transplantation. International journal of infectious diseases: IJID: official publication of the International Society for Infectious Diseases [Internet]. 2013 9 [cited 2014 Nov 30];17(9):e681–5. Available: http://www.sciencedirect.com/science/article/pii/S1201971213000726. 23490090 10.1016/j.ijid.2013.01.021

[26] Ursini T, Polilli E, Fazii P, Ieraci A, Sindici G, Parruti G. Late diagnosis of central nervous system involvement associated with lethal dissemination of Strongyloides stercoralis in an advanced HIV patient from Nigeria. International journal of infectious diseases: IJID: official publication of the International Society for Infectious Diseases [Internet]. 2013 4 [cited 2014 Nov 30];17(4):e280–2. Available: http://www.sciencedirect.com/science/article/pii/S1201971213000222. 23414737 10.1016/j.ijid.2012.11.031

[27] Carruthers VVB. Host cell invasion by the opportunistic pathogen Toxoplasma gondii. Acta tropica [Internet]. 2002 2 [cited 2014 Dec 21];81(2):111–22. Available: http://www.ncbi.nlm.nih.gov/pubmed/11801218. 11801218 10.1016/s0001-706x(01)00201-7

[28] Machala L, Malý M, Beran O, Jilich D, Kodym P. Incidence and clinical and immunological characteristics of primary Toxoplasma gondii infection in HIV-infected patients. International journal of infectious diseases: IJID: official publication of the International Society for Infectious Diseases [Internet]. 2013 10 [cited 2014 Nov 30];17(10):e892–6. Available: http://www.sciencedirect.com/science/article/pii/S120197121300146X. 23669277 10.1016/j.ijid.2013.03.017

[29] House PK, Vyas A, Sapolsky R. Predator cat odors activate sexual arousal pathways in brains of Toxoplasma gondii infected rats. PloS one [Internet]. 2011 1 [cited 2014 Nov 23];6(8):e23277 Available: http://www.pubmedcentral.nih.gov/articlerender.fcgi?artid=3157360&tool=pmcentrez&rendertype=abstract. 10.1371/journal.pone.0023277 21858053 PMC3157360

[30] De Carolis S, Santucci S, Botta a, Salvi S, Degennaro V a, Garufi C, et al The relationship between TORCH complex false positivity and obstetric outcome in patients with antiphospholipid syndrome. Lupus [Internet]. 2012 6;21(7):773–5. Available: http://www.ncbi.nlm.nih.gov/pubmed/22635229. 10.1177/0961203312447866 22635229

[31] Gonçalves MA dos S, Matos C de CB de, Spegiorin LCJF, Oliani DCMV, Oliani AH, Mattos LC de. Seropositivity rates for toxoplasmosis, rubella, syphilis, cytomegalovirus, hepatitis and HIV among pregnant women receiving care at a public health service, São Paulo state, Brazil. Brazilian Journal of Infectious Diseases [Internet]. The Brazilian Journal of Infectious Diseases and Contexto Publishing; 2010 12 [cited 2014 Oct 28];14(6):601–5. Available: http://www.scielo.br/scielo.php?script=sci_arttext&pid=S1413-86702010000600009&lng=en&nrm=iso&tlng=pt. 21340301

[32] Mattos C de CB de CC de CB de, Spegiorin LLCJF, Meira C da S, Silva T da C, Ferreira AI da C, Nakashima F, et al Anti-Toxoplasma gondii antibodies in pregnant women and their newborn infants in the region of São José do Rio Preto, São Paulo, Brazil. Sao Paulo Medical … [Internet]. Associação Paulista de Medicina; 2011 [cited 2014 Nov 3];129(4):261–6. Available: http://www.scielo.br/scielo.php?script=sci_arttext&pid=S1516-31802011000400010&lng=en&nrm=iso&tlng=en. 10.1590/S1516-31802011000400010 PMC10896019 21971902

[33] Inácio P, Costa DA, Simões MJS. Toxoplasmose em gestantes de Araraquara / SP: análise da utilização do teste de avidez de IgG anti- Toxoplasma na rotina do pré-natal. 2007;57–62.

[34] Campello Porto L, Duarte EC. Association between the risk of congenital toxoplasmosis and the classification of toxoplasmosis in pregnant women and prenatal treatment in Brazil, 1994–2009. International journal of infectious diseases: IJID: official publication of the International Society for Infectious Diseases [Internet]. 2012 7 [cited 2014 Nov 30];16(7):e480–6. Available: http://www.sciencedirect.com/science/article/pii/S1201971212000690. 22522069 10.1016/j.ijid.2012.01.016

[35] Bichara CNC, Canto GA de C, Tostes C de L, Freitas JJ da S, Carmo EL do, Póvoa MM, et al Incidence of congenital toxoplasmosis in the city of Belém, state of Pará, northern Brazil, determined by a neonatal screening program: preliminary results. Revista da Sociedade Brasileira de Medicina Tropical [Internet]. SBMT; 2012 2 [cited 2014 Dec 21];45(1):122–4. Available: http://www.scielo.br/scielo.php?script=sci_arttext&pid=S0037-86822012000100024&lng=en&nrm=iso&tlng=en. 22370842 10.1590/s0037-86822012000100024

[36] McLeod R, Boyer KM, Lee D, Mui E, Wroblewski K, Karrison T, et al Prematurity and severity are associated with Toxoplasma gondii alleles (NCCCTS, 1981–2009). Clinical infectious diseases: an official publication of the Infectious Diseases Society of America [Internet]. 2012 6 1 [cited 2014 Dec 13];54(11):1595–605. Available: http://cid.oxfordjournals.org/content/54/11/1595.short. 22499837 10.1093/cid/cis258 PMC3348955

[37] Watts P, Maguire S, Kwok T, Talabani B, Mann M, Wiener J, et al Newborn retinal hemorrhages: a systematic review. Journal of AAPOS: the official publication of the American Association for Pediatric Ophthalmology and Strabismus / American Association for Pediatric Ophthalmology and Strabismus [Internet]. American Association for Pediatric Ophthalmology and Strabismus; 2013 2 [cited 2014 Oct 17];17(1):70–8. Available: http://www.ncbi.nlm.nih.gov/pubmed/23363882. 10.1016/j.jaapos.2012.07.012 23363882

[38] Furtado JM, Lansingh VC, Carter MJ, Milanese MF, Peña BN, Ghersi H a, et al Causes of blindness and visual impairment in Latin America. Survey of ophthalmology [Internet]. 2011 [cited 2014 Dec 21];57(2):149–77. Available: http://www.ncbi.nlm.nih.gov/pubmed/22137039. 10.1016/j.survophthal.2011.07.002 22137039

[39] Toxoplasmose RB de, Toxoplasmose CC do PSN de. Letter of Buzios: Proposition for the control of toxoplasmosis in Brazil [Internet]. Scientia Medica. 2010 [cited 2014 Dec 21]. p. 5–8. Available: http://revistaseletronicas.pucrs.br/ojs/index.php/scientiamedica/article/view/6652.

[40] Nascimento LFC, Almeida MC da S, Gomes C de MS. [Neonatal mortality and avoidable causes in the micro regions of São Paulo state]. Revista brasileira de ginecologia e obstetrícia: revista da Federação Brasileira das Sociedades de Ginecologia e Obstetrícia [Internet]. 2014 7 [cited 2015 May 17];36(7):303–9. Available: http://www.ncbi.nlm.nih.gov/pubmed/25140569. 10.1590/so100-720320140005012 25140569

[41] Do Carmo Leal M, da Silva AAM, Dias MAB, da Gama SGN, Rattner D, Moreira ME, et al Birth in Brazil: national survey into labour and birth. Reproductive health [Internet]. 2012 1 [cited 2015 May 17];9(1):15 Available: http://www.reproductive-health-journal.com/content/9/1/15. 22913663 10.1186/1742-4755-9-15 PMC3500713

[42] Da Fonseca CRB, Strufaldi MWL, de Carvalho LR, Puccini RF. Adequacy of antenatal care and its relationship with low birth weight in Botucatu, São Paulo, Brazil: a case-control study. BMC pregnancy and childbirth [Internet]. 2014 1 [cited 2015 May 17];14(1):255 Available: http://www.biomedcentral.com/1471-2393/14/255. 25085236 10.1186/1471-2393-14-255 PMC4131026

[43] CDC—Toxoplasmosis. [cited 2014 Dec 21]; Available: http://www.cdc.gov/parasites/toxoplasmosis/.

[44] Hotez PJ. Neglected parasitic infections and poverty in the United States. PLoS neglected tropical diseases [Internet]. Public Library of Science; 2014 9 4 [cited 2014 Nov 11];8(9):e3012 Available: http://www.scopus.com/inward/record.url?eid=2-s2.0-84907584695&partnerID=tZOtx3y1. 10.1371/journal.pntd.0003012 25188455 PMC4154650

[45] Organization W-WH. WHO: recommended definitions, terminology and format for statistical tables related to the perinatal period and use of a new certificate for cause of perinatal deaths. Modifications recommended by FIGO as amended October 14, 1976. Acta obstetricia et gynecologica Scandinavica [Internet]. 1977 1 [cited 2014 Dec 21];56(3):247–53. Available: http://www.ncbi.nlm.nih.gov/pubmed/560099. 560099

[46] Ministério da Saúde [Internet]. [cited 2014 Dec 21]. Available: http://portal.saude.gov.br/404.html.

[47] Rodrigues A, Uezato S, Vono M, Pandossio T, Spegiorin L, Oliani A, et al Non-association between anti-Toxoplasma gondii antibodies and ABO blood group system. Journal of Venomous Animals and Toxins including Tropical Diseases [Internet]. CEVAP; 2011 [cited 2014 Dec 21];17(2):184–9. Available: http://www.scielo.br/scielo.php?pid=S1678-91992011000200009&script=sci_arttext.

[48] Porto AMF, Amorim MMR de, Coelho ICN, Santos LC. Perfil sorológico para toxoplasmose em gestantes atendidas em maternidade. Revista da Associação Médica Brasileira [Internet]. Associação Médica Brasileira; 2008 6 [cited 2014 Dec 21];54(3):242–8. Available: http://www.scielo.br/scielo.php?script=sci_arttext&pid=S0104-42302008000300018&lng=en&nrm=iso&tlng=pt. 18604403 10.1590/s0104-42302008000300018

[49] Said RN, Zaki MM, Abdelrazik MB. Congenital toxoplasmosis: evaluation of molecular and serological methods for achieving economic and early diagnosis among Egyptian preterm infants. Journal of tropical pediatrics [Internet]. 2011 10 1 [cited 2014 Nov 30];57(5):333–9. Available: http://www.ncbi.nlm.nih.gov/pubmed/20961951. 10.1093/tropej/fmq097 20961951

[50] Martins LM, Rangel ALP, Peixe RG, Silva-Dos-Santos PPSP, Lemos EM, Martins-Filho OA, et al Specific IgM, IgG and IgG1 directed against Toxoplasma gondii detected by flow cytometry and their potential as serologic tools to support clinical indirect fundoscopic presumed diagnosis of ocular disease. Journal of immunological methods [Internet]. 2015 12 16 [cited 2014 Dec 21];417:97–106. Available: http://www.ncbi.nlm.nih.gov/pubmed/25527345. 10.1016/j.jim.2014.12.012 25527345

[51] Pessanha TM, Carvalho M de, Pone MVS, Gomes SC Júnior. Abordagem diagnóstica e terapêutica da toxoplasmose em gestantes e as repercussões no recém-nascido. Revista Paulista de Pediatria [Internet]. Associação Paulista de Pediatria; 2011 9 [cited 2014 Dec 21];29(3):341–7. Available: http://www.scielo.br/scielo.php?script=sci_arttext&pid=S0103-05822011000300006&lng=en&nrm=iso&tlng=pt.

[52] Dias RCF, Lopes-Mori FMR, Mitsuka-Breganó R, Dias RAF, Tokano DV, Reiche EMV, et al Factors associated to infection by Toxoplasma gondii in pregnant women attended in Basic Health Units in the city of Rolândia, Paraná, Brazil. Revista do Instituto de Medicina Tropical de São Paulo [Internet]. Instituto de Medicina Tropical de S&atilde;o Paulo; [cited 2014 Dec 21];53(4):185–91. Available: http://www.scielo.br/scielo.php?script=sci_arttext&pid=S0036-46652011000400002&lng=en&nrm=iso&tlng=en. 21915460 10.1590/s0036-46652011000400002

[53] Ferezin RI, Bertolini DA, Demarchi IG. Prevalência de sorologia positiva para HIV, hepatite B, toxoplasmose e rubéola em gestantes do noroeste paranaense. Revista Brasileira de Ginecologia e Obstetrícia [Internet]. Federação Brasileira das Sociedades de Ginecologia e Obstetrícia; 2013 2 [cited 2014 Dec 21];35(2):66–70. Available: http://www.scielo.br/scielo.php?script=sci_arttext&pid=S0100-72032013000200005&lng=en&nrm=iso&tlng=pt. 23412005 10.1590/s0100-72032013000200005

[54] Filisetti D, Candolfi E. Immune response to Toxoplasma gondii. Ann Ist Super Sanita [Internet]. 2004 [cited 2014 Dec 31];40(1):71–80. Available: http://www.iss.it/binary/publ/publi/40171.1107854946.pdf. 15269455

[55] Sagel U, Krämer A. Screening of Maternal Toxoplasmosis in Pregnancy: Laboratory Diagnostics from the Perspective of Public Health Requirements. Journal of Bacteriology & Parasitology [Internet]. 2013 [cited 2014 Dec 21];01(S5). Available: http://pub.uni-bielefeld.de/publication/2675302.

[56] Bortoletti Filho J, Araujo E Júnior, Carvalho N da S, Helfer TM, Nogueira Serni P de O, Nardozza LMM, et al The Importance of IgG Avidity and the Polymerase Chain Reaction in Treating Toxoplasmosis during Pregnancy: Current Knowledge. Interdisciplinary perspectives on infectious diseases [Internet]. 2013 1 [cited 2015 May 17];2013:370769 Available: http://www.pubmedcentral.nih.gov/articlerender.fcgi?artid=3803120&tool=pmcentrez&rendertype=abstract. 10.1155/2013/370769 24191157 PMC3803120

[57] Inagaki AD de M, Oliveira LAR de, Oliveira MFB de, Santos RCS, Araújo RM, Alves JAB, et al Seroprevalence of antibodies for toxoplasmosis, rubella, cytomegalovirus, syphilis and HIV among pregnant women in Sergipe. Rev Soc Bras Med Trop [Internet]. [cited 2014 Dec 21];42(5):532–6. Available: http://www.scielo.br/scielo.php?script=sci_arttext&pid=S0037-86822009000500010. 19967235 10.1590/s0037-86822009000500010

[58] Lima RRCM, Amaral W do WN do, AO da C Júnior, Gonçalves FT, Tocchio ML, Cândido RL, et al Relação entre más-formações e óbitos fetais em decorrência de toxoplasmose congênita tratadas em uma clínica particular de Goiânia-GO. Ensaios e Ciência: Ciências Biológicas, Agrárias e da Saúde [Internet]. Universidade Anhanguera; 2011 [cited 2014 Dec 21];15(4):53–63. Available: http://www.sare.anhanguera.com/index.php/rensc/article/view/2012.

[59] De Souza-e-Silva CH, Vasconcelos-Santos DV, de Andrade GQ, Carellos EVM, de Castro Romanelli RM, de Resende LM, et al Association between IgG subclasses against Toxoplasma gondii and clinical signs in newborns with congenital toxoplasmosis. The Pediatric infectious disease journal [Internet]. 2013 1 [cited 2015 Jan 20];32(1):13–6. Available: http://www.ncbi.nlm.nih.gov/pubmed/22935868. 10.1097/INF.0b013e3182703460 22935868

[60] Wallon M, Peyron F, Cornu C, Vinault S, Abrahamowicz M, Kopp CB, et al Congenital toxoplasma infection: monthly prenatal screening decreases transmission rate and improves clinical outcome at age 3 years. Clinical infectious diseases: an official publication of the Infectious Diseases Society of America [Internet]. 2013 5 [cited 2014 Oct 28];56(9):1223–31. Available: http://www.ncbi.nlm.nih.gov/pubmed/23362291. 23362291 10.1093/cid/cit032

[61] Kiatchoosakun P, Suphadun W, Jirapradittha J, Yimtae K, Thanawirattananit P. Incidence and risk factors associated with hearing loss in high-risk neonates in Srinagarind Hospital. Journal of the Medical Association of Thailand = Chotmaihet thangphaet [Internet]. 2012 1 [cited 2014 Dec 31];95(1):52–7. Available: http://europepmc.org/abstract/med/22379742. 22379742

[62] Deorari A. Incidence, clinical spectrum, and outcome of intrauterine infections in neonates. Journal of Tropical Pediatrics [Internet]. 2000 6 1 [cited 2014 Nov 30];46(3):155–60. Available: http://tropej.oupjournals.org/cgi/doi/10.1093/tropej/46.3.155. 10893916 10.1093/tropej/46.3.155

[63] Romanelli RM de C, Carellos EVM, Campos FA, Pinto AS de P, Marques BA, Anchieta LM, et al The approach to neonatal congenital infections—toxoplasmosis and syphilis. Revista Médica de Minas Gerais [Internet]. 2014 [cited 2015 Jan 20];24(2):202–15. Available: http://www.rmmg.org/artigo/detalhes/1601.

[64] Pinto FS, de Andrade GMQ, Januario JN, Maia MCA, Gontijo ED. Epidemiological profile of Trypanosoma cruzi-infected mothers and live birth conditions in the state of Minas Gerais, Brazil. Revista da Sociedade Brasileira de Medicina Tropical [Internet]. Jan [cited 2015 1 20];46(2):196–9. Available: http://www.ncbi.nlm.nih.gov/pubmed/23740060. 10.1590/0037-8682-1687-2013 23740060

[65] Lopes JCCJ, Moraes MMS de, Kujumjian FG, Chiaravalloti Neto F, Lopes JCCJ. Avaliação da assistência às gestantes: o caso do município de São José do Rio Preto, São Paulo, Brasil. Rev bras saúde … [Internet]. Instituto Materno Infantil de Pernambuco; 2004 12 [cited 2015 May 17];4(4):375–84. Available: http://bvsalud.org/portal/resource/pt/lil-393386.

[66] Fricker-Hidalgo H, Cimon B, Chemla C, Darde ML, Delhaes L, L’ollivier C, et al Toxoplasma seroconversion with negative or transient immunoglobulin M in pregnant women: myth or reality? A French multicentre retrospective study. Journal of clinical microbiology [Internet]. 2013 7 [cited 2014 Dec 24];51(7):2103–11. Available: http://www.pubmedcentral.nih.gov/articlerender.fcgi?artid=3697685&tool=pmcentrez&rendertype=abstract. 10.1128/JCM.00169-13 23616461 PMC3697685

[67] McLeod R, Lykins J, Noble A, Gwendolyn Noble A, Rabiah P, Swisher CN, et al Management of Congenital Toxoplasmosis. Current Pediatrics Reports [Internet]. 2014 7 22 [cited 2015 Jan 20];2(3):166–94. Available: http://link.springer.com/article/10.1007/s40124-014-0055-7.

[68] S.Vaz R, Rauli P, Mello RG, Cardoso MA. Congenital Toxoplasmosis: A Neglected Disease?–Current Brazilian public health policy. Field Actions Science Reports [Internet]. Institut Veolia; 2011 9 1 [cited 2014 Dec 31];(Special Issue 3). Available: http://factsreports.revues.org/1086.

[69] Avelino MMM, Amaral WWN, Rodrigues IMX, Rassi AR, Gomes MBF, Costa TL, et al Congenital toxoplasmosis and prenatal care state programs. BMC infectious diseases [Internet]. 2014 1 [cited 2015 Apr 6];14(1):33 Available: http://www.biomedcentral.com/1471-2334/14/33/. 10.1186/1471-2334-14-33 PMC3918215 24438336

[70] Marcia Mussi-Pinhata M, Maria Quintana S. Screening for Infectious Diseases During Pregnancy: Which Test and Which Situation. Current Women’s Health Reviews [Internet]. Bentham Science Publishers; 2012 4 1 [cited 2015 May 21];8(2):158–71. Available: http://www.ingentaconnect.com/content/ben/cwhr/2012/00000008/00000002/art00007.

